# Does coagulopathy, anticoagulant or antithrombotic therapy matter in incisional hernia repair? Data from the Herniamed Registry

**DOI:** 10.1007/s00464-018-6127-y

**Published:** 2018-02-28

**Authors:** Ralph F. Staerkle, Henry Hoffmann, Ferdinand Köckerling, Daniela Adolf, Reinhard Bittner, Philipp Kirchhoff

**Affiliations:** 1 Department of Surgery, Clinic for Visceral Surgery, University Hospital Basel, Spitalstrasse 21, 4031 Basel, Switzerland; 2 Department of Surgery and Center for Minimally Invasive Surgery, Academic Teaching Hospital of Charité Medical School, Vivantes Hospital, Neue Bergstrasse 6, 13585 Berlin, Germany; 3 StatConsult GmbH, Halberstädter Straße 40 a, 39112 Magdeburg, Germany; 4 Winghofer Medicum Hernia Center, Winghofer Strasse 42, 72108 Rottenburg am Neckar, Germany

**Keywords:** Incisional hernia, Bleeding, Postoperative complication, Antithrombotic therapy, Anticoagulant therapy, Coagulopathy

## Abstract

**Background:**

A considerable number of patients undergoing incisional hernia repair are on anticoagulant or antiplatelet therapy or have existing coagulopathy which may put them at higher risk for postoperative bleeding complications. Data about the optimal treatment of these patients are sparse. This analysis attempts to determine the rate of postoperative bleeding complications following incisional hernia repair and the consecutive rate of reoperation among patients with coagulopathy or receiving antiplatelet and anticoagulant therapy (higher risk group) compared to patients who do not have a higher risk (normal risk group).

**Methods:**

Out of the 43,101 patients documented in the Herniamed Registry who had an incisional hernia repair, 6668 (15.5%) were on anticoagulant or antithrombotic therapy or had existing coagulopathy. The implication of that higher risk profile for onset of postoperative bleeding was investigated in multivariable analysis. Hence, other influential variables were identified.

**Results:**

The rate of postoperative bleeding in the higher risk group was 3.9% (*n* = 261) and significantly higher compared to the normal risk group at 1.6% (*n* = 564) (OR 2.001 [1.699; 2.356]; *p* < 0.001). Additionally, male gender, use of drains, larger defect size, open incisional hernia repair, lower BMI, and higher ASA score significantly increased the risk of postoperative bleeding. The rate of reoperations due to postoperative bleeding was significantly increased in the higher risk group compared to the normal risk group (2.4 vs. 1.0%; OR 1.217 [1.071; 1.382]; *p* = 0.003). Likewise, the postoperative general complication rate (6.04 vs. 3.66%; *p* < 0.001) as well as the mortality rate (0.46 vs. 0.17%; *p* < 0.001) were significantly higher in the higher risk group.

**Conclusions:**

Patients with anticoagulant or antiplatelet therapy or existing coagulopathy who undergo incisional hernia repair have a significantly higher risk for onset of postoperative bleeding. The risk of bleeding complications and complication-related reoperations seems to be lower after laparoscopic intraperitoneal onlay mesh.

**Electronic supplementary material:**

The online version of this article (10.1007/s00464-018-6127-y) contains supplementary material, which is available to authorized users.

Incisional hernias are a common finding following open abdominal surgery, with an incidence depending on size and location of the incision [1, 2]. Its incidence ranges from 3 to 20%, with even higher rates following postoperative wound infection [2]. About 50% of incisional hernias are diagnosed within the first 12 months after surgery, but they can also occur several years later, with a subsequent risk of 2% per year [2, 3].

Due to demographic trends, surgical patients may incrementally present with advanced age and associated comorbidities. Antiplatelet or anticoagulant therapy is not uncommon among these patients. Since perioperative management of patients with anticoagulation and antiplatelet medication remains challenging, a careful analysis of the individual patient situation is mandatory [4] to balance the risk of perioperative bleeding and of thromboembolic complications. Hence, in most surgical fields, awareness of postoperative bleeding complications in these patients has been successfully raised [5–7].

Recently, the problem of postoperative bleeding in hernia surgery gained more attention. Omitting antiplatelet medication prior to surgery or discontinuing oral anticoagulation therapy with heparin bridging may help to control the risk of postoperative bleeding after inguinal hernia surgery [8, 9]. However, a recent registry-based study showed a fourfold increase in postoperative bleeding following open and endoscopic inguinal hernia repair in patients with antiplatelet medication or anticoagulation [10], highlighting the need for careful perioperative anticoagulation management in hernia patients.

Postoperative hematoma formation after incisional hernia repair has generally been reported as being up to 20% in both in open and laparoscopic repair [11], requiring interventional procedures or reoperation in some cases. Since open incisional hernia repair may require more extensive dissection compared to laparoscopic repair, the risk of postoperative bleeding complications may even be higher in open cases. However, data on perioperative bleeding complications for patients with anticoagulation, coagulopathy or antiplatelet medication are not available.

Based on the data from the Herniamed Registry [12], this analysis attempts to determine the rate of postoperative bleeding complications following incisional hernia repair and the consecutive rate of bleeding-related reoperation among patients with coagulopathy or receiving antiplatelet and anticoagulant therapy compared with patients who did not have a higher risk profile. Furthermore, it aims to identify other factors, such as patient- and procedure-related factors, influencing the occurrence of bleeding complications.

## Materials and methods

The Herniamed Registry is a multicenter, internet-based hernia registry [12] into which 618 participating hospitals and surgeons in private practice (Herniamed Study Group) in Germany, Austria, and Switzerland (status: July 03, 2017) have entered data on their patients who have undergone elective hernia surgery. All patients signed an informed consent agreeing to participate. As part of the information provided to patients regarding participation in the Herniamed Quality Assurance Study and signing the informed consent declaration, all patients are informed that the treating hospital or medical practice would like to be informed about any problem occurring after the operation and that the patient has the opportunity to attend clinical examination. All postoperative complications occurring up to 30 days after surgery are recorded. On one-year follow-up, postoperative complications are once again reviewed when the general practitioner and patient complete a questionnaire.

**Fig. 1:**
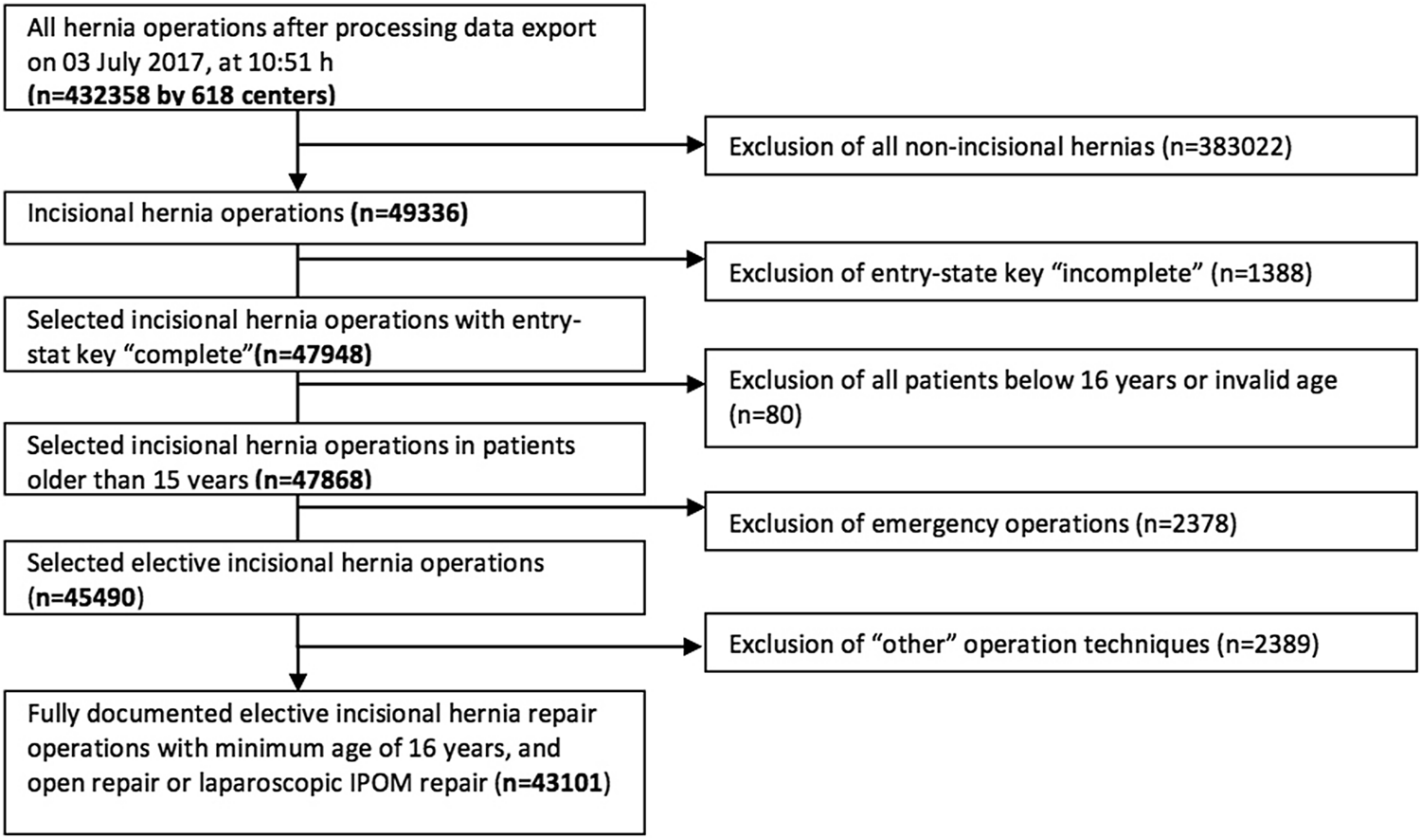
Flowchart of patient inclusion

The present analysis compares the postoperative data collected for all patients who underwent open incisional hernia repair or laparoscopic IPOM repair between September 1, 2009 and July 03, 2017. Inclusion criteria were a minimum age of 16 years, elective setting of the operation and complete registry database entry. In total, 43,101 patients were enrolled (Fig. 1). Open incisional hernia repair was performed in 29,588 of cases and the laparoscopic technique (IPOM) in 13,513 cases (Table 1).

**Table 1:** Operation techniques

Access	Technique	Coagulopathy/anticoagulant or antithrombotic therapy				Total	
		Yes		No			
		*N*	%	*N*	%	*N*	%
Open	Onlay	397	0.9	1951	4.5	2348	5.4
	Sublay	2599	6.0	12,604	29.2	15,203	35.3
	IPOM	999	2.3	5311	12.3	6310	14.6
	Component separation	162	0.4	823	1.9	985	2.3
	Direct closure	540	1.3	4202	9.7	4742	11.0
Laparoscopic	IPOM	1971	4.6	11,542	26.8	13,513	31.4
Total		6668	15.5	36,433	84.5	43,101	100.0

Frequency of coagulopathy/antithrombotic therapy for different surgical techniques

*IPOM* intraperitoneal onlay mesh

The patient group at increased risk for onset of postoperative bleeding complications was defined as that comprising patients with either existing coagulopathy (e.g., in the presence of liver cirrhosis), currently receiving platelet aggregation inhibitor therapy or who had discontinued such therapy less than seven days prior to surgery or patients whose quick or international normalized ratio (INR) was not within the normal range during the operation due to treatment with coumarin.

In the Herniamed Registry setting, no additional information, such as the product name of the platelet aggregation inhibitors, exact number of days they had been discontinued or exact INR value, was recorded.

Other potential influence factors investigated were the surgical technique, gender, American Society of Anesthesiologists (ASA) status, age, body mass index (BMI), primary versus recurrent incisional hernia, defect size (W1/W2/W3), and hernia location based on the European Hernia Society (EHS) classification [13], and drainage versus no drainage.

The outcome variable defined was postoperative bleeding within 30 days of operation. Postoperative bleeding is defined as large surface bleeding into the skin surrounding the operation area, hematomas in the operation area, major blood loss from indwelling drains, and reoperations because of postoperative bleeding.

Unadjusted analysis was carried out first to analyze an individual influence variable in respect of a target parameter. The main focus here was on the influence exerted by coagulopathy or antithrombotic therapy on increased bleeding risk. The *χ*^2^ test was used for categorical outcome variable. The robust *t* test (Satterthwaite) was used for continuous outcome variables that followed the normal distribution to analyze the influence exerted by coagulopathy or antithrombotic therapy.

A binary logistic regression model was used to study the influence of patient- (demographic) and surgery-related characteristics as well as of an increased bleeding risk associated with existing coagulopathy or antithrombotic therapy on the postoperative secondary bleeding rate, while odds ratio with 95% confidence interval was based on the Wald test. For influence variables with more than two categories, all pairwise odds ratios were provided. For the continuous influence variable “age,” the 10-year odds ratio and for the influence variable “BMI,” a five-point odds ratio was given.

In a multivariable model, several variables are analyzed for the effect on an outcome variable. Results are given as odds ratio estimates. These quantify the impact of the single variable on the outcome given that all other variables are constant. This means that estimates are adjusted for all other modeled influences. Thus, a conclusion drawn regarding variables other than the variable of interest is valid and even adjusted for by the remaining variables in the model.

## Results

Out of all patients (43,101) who had an incisional hernia repair, 6,668 (15.5%) were still receiving anticoagulant or effective antiplatelet therapy or had existing coagulopathy. Out of these, 995 (15.0%) patients had coagulopathy, *n* = 1.330 (20.0%) a Quick or INR value outside the normal range due to anticoagulation therapy, and *n* = 4831 (72.5%) of patients had treatment with platelet aggregation inhibitors which had been discontinued less than 7 days prior to surgery or had not at all been discontinued. Some patients had more than one risk factor.

### Unadjusted analysis

Unadjusted analysis of the relationship between the higher risk group (HRG) (coagulopathy, anticoagulant or antiplatelet therapy) and the normal risk group (NRG) showed that there were highly significant differences between the two groups regarding patient- and procedure-related characteristics. Patients in the HRG who were significantly older (68.8 ± 10.7 vs. 60.1 ± 14.0; *p* < 0.001) and had a (just slightly but) significantly lower BMI (29.3 ± 5.4 vs. 29.6 ± 6.1, *p* < 0.001), were predominantly male, had higher ASA scores, more open repairs, larger defect size, and more intraoperative drains (Table 2).

**Table 2:** Demographics and surgery-related parameters

	Coagulopathy/anticoagulant or antithrombotic therapy				*p*
	Yes		No		
	*n*	%	*n*	%	
Operation					
Laparoscopic	1971	29.56	11,542	31.68	< 0.001
Open	4697	70.44	24,891	68.32	
Sex					
Male	4021	60.30	17,533	48.12	< 0.001
Female	2647	39.70	18,900	51.88	
ASA score					
I	95	1.42	5057	13.88	< 0.001
II	2215	33.22	21,641	59.40	
III/IV	4358	65.36	9735	26.72	
Recurrence					
Yes	1361	20.41	7776	21.34	0.087
No	5307	79.59	28,657	78.66	
EHS classification					
Combination	567	8.50	3260	8.95	0.406
Lateral	1094	16.41	5834	16.01	
Medial	5007	75.09	27,339	75.04	
Defect size					
W1 (< 4 cm)	1953	29.29	13,737	37.70	< 0.001
W2 (≥ 4–10 cm)	3301	49.51	16,546	45.41	
W3 (≥ 10 cm)	1414	21.21	6150	16.88	
Drainage					
Yes	3959	59.37	18,957	52.03	< 0.001
No	2709	40.63	17,476	47.97	

Unadjusted analysis for demographics and surgery-related parameters

*ASA* American Society of Anesthesiologists, *EHS* European Hernia Society

As shown in unadjusted analyses in Table 3, the overall postoperative surgical complication rate in the HRG was significantly higher compared to the NRG (12.0 vs. 7.6%, *p* < 0.001). Bowel injury, ileus, bleeding, seroma, wound healing disorders and deep infections were significantly more frequent in the HRG. Postoperative bleeding occurred significantly more frequently in the HRG (3.9 vs. 1.6%, *p* < 0.001) (Table 3).

**Table 3:** Postoperative surgical complications following incisional hernia repair

	Coagulopathy/anticoagulant or antithrombotic therapy				*p* value
	Yes		No		
	*n*	%	*n*	%	
Postoperative complications					
Yes	797	11.95	2762	7.58	< 0.001
No	5871	88.05	33,671	92.42	
Bowel injury					
Yes	51	0.76	153	0.42	< 0.001
No	6617	99.24	36,280	99.58	
Ileus					
Yes	66	0.99	213	0.58	< 0.001
No	6602	99.01	36,220	99.42	
Deep infection					
Yes	101	1.51	438	1.20	0.035
No	6567	98.49	35,995	98.80	
Bleeding					
Yes	261	3.91	564	1.55	< 0.001
No	6407	96.09	35,869	98.45	
Seroma					
Yes	303	4.54	1272	3.49	< 0.001
No	6365	95.46	35,161	96.51	
Wound healing disorder					
Yes	200	3.00	734	2.01	< 0.001
No	6468	97.00	35,699	97.99	

The overall complication-related reoperation rate in the HRG was significantly higher compared to the NRG (5.7 vs. 3.6%; *p* < 0.001). The postoperative bleeding-related reoperation rate was also significantly higher in the HRG (2.4 vs. 1.0%; *p* < 0.001) (Table 4).

**Table 4:** Postoperative complication-related reoperation rates

	Coagulopathy/anticoagulant or antithrombotic therapy				*p*
	Yes		No		
	*n*	%	*n*	%	
Postoperative bleeding					
Yes	261	3.91	564	1.55	< 0.001
No	6407	96.09	35,869	98.45	
Complication-related reoperation rate					
Yes	377	5.65	1303	3.58	< 0.001
No	6291	94.35	35,130	96.42	
Bleeding-related reoperation rate					
Yes	163	2.44	356	0.98	< 0.001
No	6505	97.56	36,077	99.02	

Unadjusted analysis for postoperative bleeding and reoperation rates (overall and bleeding related)

No significant difference was found between the intraoperative complication rates (HRG 2.25% vs. NRG 1.92%; *p* = 0.074). However, significant differences were also identified in the postoperative general complication rates to the disadvantage of the HRG (6.04 vs. 3.66%; *p* < 0.001). Likewise, the mortality rate in the HRG was significantly higher (0.46 vs. 0.17%; *p* < 0.001).

### Differences in the subgroups: unadjusted analysis

An additional analysis was performed to identify whether there were any differences in the postoperative bleeding rates and in the bleeding-related reoperation rates between the coagulopathy, anticoagulant therapy and antiplatelet therapy subgroups. To that effect, the groups with only one risk factor were compared with each other (total: *n* = 6210, coagulopathy: *n* = 664, antiplatelet therapy: *n* = 4506, anticoagulant therapy: *n* = 1040). Significant differences were found in the postoperative bleeding rates of 5.3% for the coagulopathy subgroup, 7.5% for the anticoagulant therapy subgroup, and 2.5% for the antiplatelet therapy subgroup (*p* < 0.001). Accordingly, significant differences were also detected in the bleeding-related reoperation rates (3.8 vs. 4.8 vs. 1.4%; *p* < 0.001). As such, the risk of both postoperative bleeding and bleeding-related reoperation was lowest in the antiplatelet therapy patient group.

### Multivariable analysis of postoperative bleeding

The results of the multivariable analysis of postoperative bleeding are summarized in Table 5 (model fit *p* < 0.001). The risk of postoperative bleeding in open and laparoscopic cases is significantly higher in patients with coagulopathy, anticoagulant or antiplatelet therapy (*p* < 0.001) with an odds ratio of 2.001 [1.699; 2.356]. There were significantly more cases of postoperative bleeding in the group with drains (OR 2.016 [1.653; 2.457]; *p* < 0.001), in male patients (OR 1.525 [1.319; 1.763]; *p* < 0.001), in larger defects (e.g., W3 vs. W1: *OR* 1.683 [1.360; 2.083]; *p* < 0.001), and after open repair (OR 1.644 [1.317; 2.052]; *p* < 0.001). In the higher ASA categories (ASA III/IV vs. II: OR 1.244 [1.064; 1.455]; *p* = 0.006; ASA III/IV vs. I: OR 1.524 [1.114; 2.084]; *p* = 0.008), the risk of postoperative bleeding was increased as well. In contrast, the risk of postoperative bleeding decreases with higher BMI (five-point BMI: *OR* 0.895 [0.837; 0.956]; *p* < 0.001).

**Table 5:** Multivariable analysis of postoperative bleeding following open and laparoscopic incisional hernia repair

Parameter	*p* value	Category	Paired *p* value	OR	[95% CI]	
Coagulopathy, anticoagulant, or antithrombotic therapy	< 0.001	Yes vs. no	< 0.001	2.001	1.699	2.356
Drainage	< 0.001	Yes vs. no	< 0.001	2.016	1.653	2.457
Gender	< 0.001	Male vs. female	< 0.001	1.525	1.319	1.763
Defect size	< 0.001	W3 (≥ 10 cm) vs. W2 (≥ 4–10 cm)	0.053	1.180	0.998	1.396
		W3 (≥ 10 cm) vs. W1 (< 4 cm)	< 0.001	1.683	1.360	2.083
		W2 (≥ 4–10 cm) vs. W1 (< 4 cm)	< 0.001	1.426	1.185	1.717
Operation technique	< 0.001	Open vs. laparoscopic	< 0.001	1.644	1.317	2.052
BMI [5-point OR]	< 0.001			0.895	0.837	0.956
ASA	0.005	III/IV vs. II	0.006	1.244	1.064	1.455
		III/IV vs. I	0.008	1.524	1.114	2.084
		II vs. I	0.177	1.225	0.912	1.644
EHS classification	0.057	Medial vs. combined	0.413	1.108	0.867	1.417
		Medial vs. lateral	0.020	1.283	1.040	1.583
		Combined vs. lateral	0.345	1.158	0.854	1.570
Age [10-year OR]	0.282			1.033	0.973	1.097
Recurrences	0.645	Yes vs. no	0.645	1.041	0.878	1.233

### Multivariable analysis of reoperations due to postoperative bleeding

The results of the multivariable analysis of reoperations due to postoperative bleeding are summarized in Table 6 (model fit *p* < 0.001). The use of drains (OR 2.714 [2.082; 3.539]; *p* < 0.001), coagulopathy, antiplatelet or anticoagulant therapy (OR 1.944 [1.584; 2.387]; *p* < 0.001), male gender (OR 1.629 [1.356; 1.957]; *p* < 0.001), larger defect size (W3 vs. W1: OR 1.745 [1.330; 2.291]; *p* < 0.001 W2 vs. W1: OR 1.535 [1.209; 1.947]; *p* < 0.001), open incisional hernia repair (OR 1.482 [1.114; 1.970]; *p* = 0.007), and higher ASA scores (ASA III/IV vs. II: OR 1.320 [1.085; 1.606]; *p* = 0.006 ASA III/IV vs. I: OR 1.531 [1.043; 2.247]; *p* = 0.030) increased the risk of reoperation due to postoperative bleeding significantly. In contrast, the risk of reoperation due to postoperative bleeding decreases with higher BMI (five-point BMI OR 0.817 [0.750; 0.890]; *p* < 0.001) (Table 6).

**Table 6:** Multivariable analysis of reoperations due to postoperative secondary bleeding

Parameter	*p* value	Category	Paired *p* value	OR	95% CI	
Drainage	< 0.001	Yes vs. no	< 0.001	2.714	2.082	3.539
Coagulopathy, antithrombotic therapy	< 0.001	Yes vs. no	< 0.001	1.944	1.584	2.387
Gender	< 0.001	Male vs. female	< 0.001	1.629	1.356	1.957
BMI [5-point OR]	< 0.001			0.817	0.750	0.890
Defect size	< 0.001	W3 (≥ 10 cm) vs. W2 (≥ 4–10 cm)	0.226	1.137	0.923	1.401
		W3 (≥ 10 cm) vs. W1 (< 4 cm)	< 0.001	1.745	1.330	2.291
		W2 (≥ 4–10 cm) vs. W1 (< 4 cm)	< 0.001	1.535	1.209	1.947
Operation technique	0.007	Open vs. laparoscopic	0.007	1.482	1.114	1.970
ASA	0.010	III/IV vs. II	0.006	1.320	1.085	1.606
		III/IV vs. I	0.030	1.531	1.043	2.247
		II vs. I	0.420	1.160	0.809	1.663
EHS classification	0.065	Medial vs. combined	0.393	1.145	0.840	1.560
		Medial vs. lateral	0.024	1.365	1.042	1.789
		Combined vs. Lateral	0.373	1.193	0.809	1.759
Recurrence	0.222	Yes vs. no	0.222	1.139	0.925	1.403
Age [10-year OR]	0.777			0.989	0.919	1.065

### Multivariable analysis of reoperations due to postoperative complications

The results of the multivariable analysis of reoperations due to postoperative complications are summarized in Table 7 (model fit *p* < 0.001). The risk of reoperation is significantly associated with larger defects (W3 vs. W2: OR 1.611 [1.439; 1.803]; *p* < 0.001; W3 vs. W1: OR 2.690 [2.306; 3.139]; *p* < 0.001; W2 vs. W1: OR 1.670 [1.449; 1.925]; *p* < 0.001), with the use of drains (OR 1.986 [1.720; 2.292]; *p* < 0.001), higher ASA scores (ASA III/IV vs. II: OR 1.525 [1.366; 1.703]; p < 0.001; ASA III/IV vs. I: OR 1.636 [1.318; 2.032]; *p* < 0.001), open surgery (OR 1.618 [1.385; 1.892]; *p* < 0.001), recurrent incisional hernias (OR 1.252 [1.117; 1.403]; *p* < 0.001), and EHS classification (*p* < 0.001) in terms of medial compared to lateral incisional hernias (OR 1.394 [1.192; 1.629]; *p* < 0.001), and combined compared to lateral incisional hernias (OR 1.359 [1.097; 1.682]; *p* = 0.005). Existing coagulopathy, anticoagulant or antiplatelet therapy (OR 1.217 [1.071; 1.382]; *p* = 0.003) and patients with a higher BMI (five-point BMI: OR 1.065 [1.021; 1.110]; *p* = 0.003) increase the risk of reoperation (Table 7).

**Table 7:** Multivariable analysis of reoperations due to postoperative complications

Parameter	*p* value	Category	Paired *p* value	OR	95% CI	
Defect size	< 0.001	W3 (≥ 10 cm) vs. W2 (≥ 4–10 cm)	< 0.001	1.611	1.439	1.803
		W3 (≥ 10 cm) vs. W1 (< 4 cm)	< 0.001	2.690	2.306	3.139
		W2 (≥ 4–10 cm) vs. W1 (< 4 cm)	< 0.001	1.670	1.449	1.925
Drainage	< 0.001	Yes vs. no	< 0.001	1.986	1.720	2.292
ASA	< 0.001	III/IV vs. II	< 0.001	1.525	1.366	1.703
		III/IV vs. I	< 0.001	1.636	1.318	2.032
		II vs. I	0.503	1.073	0.873	1.317
Operation technique	< 0.001	Open vs. laparoscopic	< 0.001	1.618	1.385	1.892
Recurrence	< 0.001	Yes vs. no	< 0.001	1.252	1.117	1.403
EHS classification	< 0.001	Medial vs. combined	0.764	1.026	0.869	1.210
		Medial vs. lateral	< 0.001	1.394	1.192	1.629
		Combined vs. Lateral	0.005	1.359	1.097	1.682
Coagulopathy, antithrombotic therapy	0.003	Yes vs. no	0.003	1.217	1.071	1.382
BMI [5-point OR]	0.003			1.065	1.021	1.110
Alter [10-year OR]	0.150			1.032	0.989	1.076
Sex	0.202	Male vs. female	0.202	1.068	0.965	1.181

## Discussion

Our study showed that patients undergoing incisional hernia repair with existing coagulopathy, anticoagulant or antiplatelet therapy have a significantly higher postoperative surgical complication rate, postoperative bleeding rate, postoperative general complication rate, and mortality compared to patients without these pre-existing conditions. The risk of bleeding or of bleeding-related reoperation was significantly lower for the antiplatelet therapy subgroup than for the coagulopathy and anticoagulant therapy subgroups. Comparing open and laparoscopic IPOM approach, the postoperative bleeding rate was higher in open repair compared to laparoscopic IPOM repair, with significantly higher rates of postoperative bleeding in the HRG. Accordingly, the overall reoperation rate and bleeding-related reoperation rate were both significantly higher after open repair compared to laparoscopic repair. Since the registry does not record any information on this, the extent of dissection in laparoscopic IPOM cases is unknown. In particular, it remains unclear whether all connective and fatty tissues were removed from the “landing zone” in the region of the round ligament of liver (hepatic teres ligament) and the hepatic falciform ligament.

Another inherent weakness of a registry study is the fact that outcome criteria for bleeding seem very subjective. Bleeding on the skin or the volume of blood in the drain is not specified, nor is the person reporting this identified.

The multivariable analysis revealed other factors, which are significantly associated with an increased risk of postoperative bleeding, such as the use of drains, male gender, larger hernia defect size, and higher ASA score. Although there is a higher risk of bleeding in large defects, multivariable analysis shows an additional significant effect of the technique.

Furthermore, the reoperation rate due to bleeding-related complications as well as the reoperation rate due to other complications was significantly higher in the HRG compared to the NRG.

Postoperative bleeding and consecutive hematomas are bothersome for the patients and have a strong clinical impact requiring interventional or operative treatment [14]. Postoperative bleeding is a typical adverse event occurring after surgery [15]. Regarding inguinal hernia surgery alone, postoperative bleeding is the most frequent adverse event [16, 17]. A recent registry-based analysis of 82,911 patients undergoing open or endoscopic [transabdominal preperitoneal patch plasty (TAPP), total extraperitoneal patch plasty (TEP)] inguinal hernia repair showed a fourfold higher risk for onset of postoperative bleeding in patients with existing anticoagulation or antithrombotic therapy [10]. Surprisingly, the endoscopic procedures (TAPP, TEP) showed lower postoperative bleeding rates compared to open inguinal hernia repair, although the endoscopic techniques were deemed as being more likely to cause postoperative bleeding due to their more extensive tissue dissection. Additionally, larger hernia defect size, male gender, higher ASA score, and recurrent operation were identified as significant risk factors for postoperative bleeding in the registry population [10]. However, data on the risk of postoperative bleeding among patients undergoing incisional hernia repair are rare. Usually, incisional hernia repair is an elective procedure with carefully prepared patients. Since incisional hernias can present with larger defect sizes posing additional challenges, such as loss of domain, previous abdominal surgery or intraabdominal adhesions, prevention of postoperative bleeding necessitating reoperation seems to be of utmost importance. This is even more important in view of the fact that postoperative bleeding complications are deemed to be risk factors for recurrence after laparoscopic ventral hernia repair [18].

A recent study using a propensity score analysis of 486 consecutive patients undergoing incisional hernia repair revealed anticoagulation as a pre-existing condition frequently found in the risk group for postoperative bleeding, developing hematomas in 9.9% of open cases and 3.3% of laparoscopic IPOM cases [19]. In our study, however, we demonstrated that postoperative bleeding occurred in up to 3.9% of patients in the HRG of patients with coagulopathy, anticoagulant or antithrombotic therapy, leading to a significantly higher bleeding-related reoperation rate of 2.44% (*n* = 163) in the HRG compared to the NRG (0.98%). This demonstrates that coagulopathy, anticoagulant or antithrombotic therapy is an evident risk factor for postoperative bleeding requiring reoperation in a reasonable number of patients, with known unfavorable consequences such as prolonged hospital stays and increased direct and indirect healthcare costs.

The type of surgical approach in hernia surgery and its impact on postoperative complications are an ongoing debate. A recent analysis revealed substantial variation among hernia experts regarding decision-making in treatment strategies for incisional hernia patients [20], highlighting the difficulties in selecting the most appropriate surgical technique for the patient. Thus, it must be assumed that to date the choice of surgical technique in incisional hernia repair has been influenced more by the hernia parameters than by individual patient factors. Our study results demonstrate that the choice of surgical technique has significant impact on the postoperative outcome.

Apart from the surgical technique, a larger defect size, male gender, and higher ASA classification, intraoperative drains were identified as further risk factors for occurrence of postoperative bleeding. However, intraoperative drains must be viewed as a false-positive risk factor since drains themselves are unlikely to cause postoperative bleeding. It can be assumed that most surgeons usually place drains when they suspect possible postoperative bleeding, such as in high-risk patients.

Our data show that patients with abnormal INR or inadequate discontinuation of their antiplatelet or anticoagulation therapy undergoing incisional hernia repair are a high-risk population for onset of postoperative bleeding. Surgery for patients with these pre-existing conditions should be postponed or patients should be assigned to laparoscopic surgery when technically feasible and surgically meaningful, since laparoscopic IPOM repair has been shown to provide much more favorable outcomes compared to open repair. However, the advantages of the laparoscopic IPOM method should be carefully balanced against the described, technically inherent complications and the concerns arising from the emerging discussion of IPOM-related mesh complications in the abdominal cavity [21–23].

## Electronic supplementary material

Below is the link to the electronic supplementary material.

Supplementary material 1 (DOCX 168 KB)
