# Supplementary material for: Does coagulopathy, anticoagulant or antithrombotic therapy matter in incisional hernia repair? Data from the Herniamed Registry
Source: Surg Endosc. 2018 Feb 28;32(9):3881–9. doi: 10.1007/s00464-018-6127-y (PMC6096530; doi:10.1007/s00464-018-6127-y)
Supplement: Supplementary file 1 — Supplementary material 1 (DOCX 168 KB) [file 464_2018_6127_MOESM1_ESM.docx]

**Appendix: Herniamed Study Group**

**Scientific Board:**

**Köckerling**, Ferdinand (Chairman) (Berlin); **Bittner**, Reinhard (Rottenburg); **Fortelny**, René (Wien); **Jacob**, Dietmar (Berlin); **Koch**, Andreas (Cottbus); **Kraft,** Barbara (Stuttgart); **Kuthe**, Andreas (Hannover); **Lammers**, Bernhard (Neuss); **Lippert**, Hans (Magdeburg): **Lorenz**, Ralph (Berlin); **Mayer**, Franz (Salzburg); **Niebuhr**, Henning (Hamburg); **Peiper**, Christian (Hamm); **Pross**, Matthias (Berlin); **Reinpold**, Wolfgang (Hamburg); **Simon**, Thomas (Weinheim); **Stechemesser**, Bernd (Köln); **Unger**, Solveig (Chemnitz), **Weyhe**, Dirk (Oldenburg); **Zarras**, Konstantinos (Düsseldorf)

**Paticipants:**

**Ahmetov**, Azat (Saint-Petersburg); **Alapatt,** Terence Francis (Frankfurt/Main); **Albayrak**, Nurretin (Herne); **Amann**, Stefan (Neuendettelsau); **Anders,** Stefan (Berlin); **Anderson**, Jürina (Würzburg); **Antoine**, Dirk (Leverkusen); **Apfelstedt**, Heinrich (Solingen); **Arndt**, Anatoli (Elmshorn); **Aschenbrenner**, Michael (Spittal/Drau); **Asperger,** Walter (Halle); **Avram**, Iulian (Saarbrücken); **Baikoglu-Endres**, Corc (Weißenburg i. Bay.); **Bandowsky**, Boris (Damme); **Barkus**; Jörg (Velbert); **Becker**, Matthias (Freital); **Behrend**, Matthias (Deggendorf); **Berkhoff,** Christian (Fulda); **Beuleke,** Andrea (Burgwedel); **Birk**, Dieter (Bietigheim-Bissingen); **Bittner,** Reinhard (Rottenburg); **Blaha,** Pavel (Zwiesel); **Blumberg,** Claus (Lübeck); **Böckmann,** Ulrich (Papenburg); **Böhle**, Arnd Steffen (Bremen); **Bolle**, Ludger (Berlin); **Borchert,** Erika (Grevenbroich); **Born**, Henry (Leipzig); **Brabender,** Jan (Köln); **Breitenbuch von**, Philipp (Radebeul); **Brož**, Miroslav (Ebersbach); **Brückner**, Torsten (Gießen); **Brütting**, Alfred (Erlangen); **Buchert**, Annette (Mallersdorf-Pfaffenberg; **Buchholz,** Torsten (Aurich); **Budzier**, Eckhard (Meldorf); **Burchett**, Bert (Teterow); **Burghardt**, Jens (Berlin); **Cejnar**, Stephan-Alexander (München); **Chirikov**, Ruslan (Dorsten); **Claußnitzer**, Christian (Ulm); **Comman,** Andreas (Bogen); **Crescent**i, Fabio (Verden/Aller); **Daniels**, Thies (Hamburg); **Dapunt**, Emanuela (Bruneck); **Decker**, Georg (Berlin); **Demmel**, Michael (Arnsberg); **Descloux,** Alexandre (Baden); **Deusch**, Klaus-Peter (Wiesbaden); **Dick**, Marcus (Neumünster); **Dieterich**, Klaus (Ditzingen); **Dietz**, Harald (Landshut); **Dittmann**, Michael (Northeim); **Dominguez**, Emilio (Lichtenfels); **Drummer**, Bernhard (Forchheim); **Eckermann**, Oliver (Luckenwalde); **Eckhoff,** Jörn /Hamburg); **Ehmann**, Frank (Grünstadt); **Eisenkrein**, Alexander (Düren); **Elger**, Karlheinz (Germersheim); **Engelhardt**, Thomas (Erfurt); **Erichsen,** Axel (Friedrichshafen); **Eucker**, Dietmar (Bruderholz); **Fackeldey**, Volker (Kitzingen); **Faddah**, Yousif (Kamenz); Farke, Stefan (Delmenhorst); **Faust**, Hendrik (Emden); **Federmann**, Georg (Seehausen); **Fiedler,** Michael (Eisenberg); **Fikatas**, Panagiotis (Berlin); **Firl**, Michaela (Perleberg); **Fischer**, Ines (Wiener Neustadt); **Fleischer**, Sabine (Dinslaken); **Fortelny**, René H. (Wien); **Franczak**, Andreas (Wien); **Franke**, Claus (Düsseldorf); **Frankenberg von**, Moritz (Salem); **Frehner**, Wolfgang (Ottobeuren); **Friedhoff**, Klaus (Attendorn); **Friedrich,** Jürgen (Essen); **Frings**, Wolfram (Bonn); **Fritsche**, Ralf (Darmstadt); **Frommhold,** Klaus (Coesfeld); **Frunder**, Albrecht (Tübingen); **Fuhrer**, Günther (Reutlingen); **Garlipp**, Ulrich (Bitterfeld-Wolfen); **Gassler,** Harald (Villach); **Gawad**, Karim A. Frankfurt/Main); **Gehrig**, Tobias (Sinsheim); **Gerdes**, Martin (Ostercappeln); **Germanov**, German (Halberstadt; **Gilg**, Kai-Uwe (Hartmannsdorf); **Glaubitz**, Martin (Neumünster); **Glauner-Goldschmidt**, Kerstin (Werne); **Glutig,** Holger (Meissen); **Gmeiner**, Dietmar (Bad Dürrnberg); **Göring**, Herbert (München); **Grebe**, Werner (Rheda-Wiedenbrück); **Grothe**, Dirk (Melle); **Günther**, Thomas (Dresden); **Gürtler**, Thomas (Zürich); **Hache**, Helmer (Löbau); **Hämmerle**, Alexander (Bad Pyrmont); **Haffner**, Eugen (Hamm); **Hain**, Hans-Jürgen (Gross-Umstadt); **Halter**, Christian Jörn (Recklinghausen); **Hammans**, Sebastian (Lingen); **Hampe**, Carsten (Garbsen); **Hanke**, Stefan (Halle); **Harrer**, Petra (Starnberg); **Hartung**, Peter (Werne); **Heinzmann**, Bernd (Magdeburg); **Heise**, Joachim Wilfried (Stolberg); **Heitland**, Tim (München); **Helbling**, Christian (Uznach/Schweiz); **Hellinger**, Achim (Fulda); **Hempen**, Hans-Günther (Cloppenburg); **Hennes**, Norbert (Duisburg); **Herdter,** Christian (Gelsenkirchen); **Hermes**, Wolfgang (Weyhe); **Herzing**, Holger Höchstadt); **Hessler**, Christian (Bingen); **Heuer**, Matthias (Herten); **Hildebrand**, Christiaan (Langenfeld); **Höferlin**, Andreas (Mainz); **Hoffmann**, Henry (Basel); **Hoffmann**, Michael (Kassel); **Hofmann**, Eva M. (Frankfurt/Main); **Horbach**, Thomas (Fürth); **Hornung**, Frederic (Wolfratshausen); **Hudak**, Attila (Suhl); **Hübel-Abe**, Jan (Ilmenau; **Hügel**, Omar (Hannover); **Hüttemann**, Martin (Oberhausen); **Hüttenhain**, Thomas (Mosbach); **Hunkeler**, Rolf (Zürich); **Imdahl**, Andreas (Heidenheim); **Iseke**, Udo (Duderstadt); **Isemer**, Friedrich-Eckart (Wiesbaden); **Jablonski**, Herbert Gustav (Sögel); **Jacob**, Dietmar (Berlin); **Jansen-Winkeln**, Boris (Leipig); **Jantschulev,** Methodi (Waren); **Jenert**, Burghard (Lichtenstein); **Jugenheimer**, Michael (Herrenberg); **Junge**, Karsten (Aachen); **Kaaden**, Stephan (Neustadt am Rübenberge); **Käs**, Stephan (Weiden); **Kahraman,** Orhan (Hamburg); **Kaiser,** Christian (Westerstede); **Kaiser**, Gernot Maximilian (Kamp-Lintfort); **Kaiser**, Stefan (Kleinmachnow); **Karch**, Matthias (Eichstätt); **Kasparek**, Michael S. (München); **Kastl**, Sigrid (Braunau am Inn); **Keck**, Heinrich (Wolfenbüttel); **Keller,** Hans W. (Bonn); **Kewer**, Jans Ludolf (Tuttlingen); **Kienzle**, Ulrich (Karlsruhe); **Kipfmüller**, Brigitte (Köthen); **Kirsch**, Ulrike (Oranienburg); **Klammer**, Frank (Ahlen); **Klatt**, Richard (Hagen); **Kleeff**, Jörg (Halle/Saale); **Klein**, Karl-Hermann (Burbach); **Kleist**, Sven (Berlin); **Klobusicky**, Pavol (Bad Kissingen); **Kneifel**, Thomas (Datteln); **Knolle**, Winfried (Pritzwalk); **Knoop**, Michael (Frankfurt/Oder); **Knotter**, Bianca (Mannheim); **Koch,** Andreas (Cottbus); **Koch**, Andreas (Münster); Köckerling**,** Ferdinand (Berlin); **Köhler**, Gernot (Linz); **König**, Oliver (Buchholz); **Kornblum**, Hans (Tübingen); **Krämer**, Dirk (Bad Zwischenahn); **Kraft**, Barbara (Stuttgart); **Kratsch**, Barthel (Dierdorf/Selters); **Krausbeck**, Matthias (Schwerin); **Kreissl**, Peter (Ebersberg); **Krones**, Carsten Johannes (Aachen); **Kronhardt**, Heinrich (Neustadt am Rübenberge); **Krückel**, Karl-Heinz (Alsdorf); **Kruse,** Christinan (Aschaffenburg); **Kube**, Rainer (Cottbus); **Kühlberg**, Thomas (Berlin); **Kühn**, Gert (Freiberg); **Kuhn,** Roger (Gifhorn); **Kusch,** Eduard (Gütersloh); **Kuthe,** Andreas (Hannover); **Ladberg**, Ralf (Bremen); **Ladra**, Jürgen (Düren); **Lahr-Eigen**, Rolf (Potsdam); **Lainka**, Martin (Wattenscheid); **Lalla**, Thomas (Oschersleben); **Lammers**, Bernhard J. (Neuss); **Lancee**, Steffen (Alsfeld); **Lange**, Claas (Ludwigsfelde); **Langer**, Claus (Göttingen); **Laps**, Rainer (Ehringshausen); **Larusson**, Hannes Jon (Pinneberg); **Lauschke**, Holger (Duisburg); **Lechner-Puschnig**, Marina (Klagenfurt am Wörthersee/Österreich); **Leher,** Markus (Schärding); **Leidl**, Stefan (Waidhofen/Ybbs); **Leisten**, Edith (Köln); **Lenz**, Stefan (Berlin); **Liedke**, Marc Olaf (Heide); **Lienert**, Mark (Duisburg); **Limberger**, Andreas (Schrobenhausen); **Limmer**, Stefan (Würzburg); **Locher**, Martin (Kiel); **Loghmanieh**, Siawasch (Viersen); **Lorenz**, Ralph (Berlin); **Luedtke**, Clinton (Kusel); **Luther**, Stefan (Wipperfürth); **Luyken**, Walter (Sulzbach-Rosenberg); **Mallmann**, Bernhard (Krefeld); **Manger**, Regina (Schwabmünchen); **Markus** Peter M. (Essen); **Maurer**, Stephan (Münster); **May**, Jens Peter (Schönebeck); **Mayer**, Franz (Salzburg); **Mayer**, Jens (Schwäbisch Gmünd); **Mellert**, Joachim (Höxter); **Menzel**, Ingo (Weimar); **Meurer**, Kirsten (Bochum); **Meyer**, Moritz (Ahaus**)**; **Mirow**, Lutz (Zwickau); **Mittag-Bonsch**, Martina (Crailsheim); **Möbius**, Ekkehard (Braunschweig), **Mörder-Köttgen**, Anja (Freiburg); **Moesta**, Kurt Thomas (Hannover); **Mugomba**, Gilbert (Dannenberg); **Moldenhauer**, Ingolf (Braunschweig); **Morkramer**, Rolf (Radevormwald); **Mosa**, Tawfik (Merseburg); **Müller**, Volker (Nürnberg); **Münzberg**, Gregor (Berlin); **Murr**, Alfons (Vilshofen); **Nartschik**, Peter (Quedlinburg); **Nasifoglu**, Bernd (Ehingen); **Neumann**, Jürgen (Haan); **Neumeuer**, Kai (Paderborn); **Niebuhr,** Henning (Hamburg); **Nix**, Carsten (Walsrode); **Nölling**, Anke (Burbach); **Nostitz**, Friedrich Zoltán (Mühlhausen); **Nussbaumer**, Peter (Lachen); **Obermaier**, Straubing); **Öz-Schmidt**, Meryem (Hanau); **Olivieri**, Manuel (Pforzheim); **Passon**, Marius (Freudenberg); **Pein**, Tobias (Hameln); **Peiper**, Christian (Hamm); **Pertl**, Alexander (Spittal/Drau); **Philipp**, Mark (Rostock); **Pickart**, Lutz (Bad Langensalza); **Pizzera**, Christian (Graz); **Pöllath**, Martin (Sulzbach-Rosenberg); **Pöschmann**, Enrico (Thalwil); **Possin**, Ulrich (Laatzen); **Prenzel**, Klaus (Bad Neuenahr-Ahrweiler); **Pröve**, Florian (Goslar); **Pronnet**, Thomas (Fürstenfeldbruck); **Pross**, Matthias (Berlin); **Puff**, Johannes (Dinkelsbühl); **Rabl**, Anton (Passau); **Raggi**, Matthias Claudius (Stuttgart); **Rapp**, Martin (Neunkirchen); **Reck**, Thomas (Püttlingen); **Reinpold,** Wolfgang (Hamburg); **Renter**, Marc Alexander (Moers); **Reuter**, Christoph (Quakenbrück); **Radke,** Alexander (Thun/Zweisimmen); **Radzwewitz**, Bernd (Bergen); (**Richter,** Jörg (Winnenden); **Riemann**, Kerstin (Alzenau-Wasserlos); **Riesener**, Klaus-Peter (Marl); **Rodehorst**, Anette (Otterndorf); **Roehr**, Thomas (Rödental); **Rössler**, Michael (Rüdesheim am Rhein); **Roncossek**, Bremerhaven); **Rosniatowski**, Rolland (Marburg); **Roth** Hartmut (Nürnberg); **Sardoschau**, Nihad (Saarbrücken); **Sauer**, Gottfried (Rüsselsheim); **Sauer**, Jörg (Arnsberg); **Seekamp**, Axel (Freiburg); **Seelig**, Matthias (Bad Soden); **Seidel**, Hanka (Eschweiler); **Seiler**, Christoph Michael (Warendorf); **Seltmann,** Cornelia (Hachenburg); **Senkal,** Metin (Witten); **Shamiyeh**, Andreas (Linz); **Shang**, Edward (München); **Sieblist**, Frank (Hanau); **Siemssen**, Björn (Berlin); **Sievers,** Dörte (Hamburg); **Silbernik**, Daniel (Bonn); **Simon**, Thomas (Weinheim); **Sinn**, Daniel (Olpe); **Sinner**, Guy (Merzig); **Sinning**, Frank (Nürnberg); **Smaxwil**, Constatin Aurel (Stuttgart); **Sörensen**, Björn (Lauf an der Pegnitz): **Sucke**, Jochen Markus (Gießen); **Syga**, Günter (Bayreuth); **Schabel**, Volker (Kirchheim/Teck); **Schadd**, Peter (Euskirchen); **Schassen von**, Christian (Hamburg); **Schattenhofer**, Thomas (Vilshofen); **Scheibel**, Mike (Krefeld); **Schelp**, Lothar (Wuppertal); **Scherf**, Alexander (Pforzheim); **Scheuerlein**, Hubert (Paderborn); **Schilling**, André (Kamen); **Schimmelpenning,** Hendrik (Neustadt in Holstein); **Schinkel**, Svenja (Kempten); **Schmid**, Michael (Gera); **Schmid,** Thomas (Innsbruck); **Schmidt,** Ulf (Mechernich); **Schmitz**, Heiner (Jena); **Schmitz**, Ronald (Altenburg); **Schöche**, Jan (Borna); **Schoenen**, Detlef (Schwandorf); **Schrittwieser**, Rudolf (Bruck an der Mur); **Schroll**, Andreas (München); **Schubert**, Daniel (Saarbrücken); **Schüder**, Gerhard (Wertheim); **Schürmann**, Rainer (Steinfurt); **Schultz**, Christian (Bremen-Lesum); **Schultz**, Harald (Landstuhl); **Schulze**, Frank P. Mülheim an der Ruhr); **Schulze**, Thomas (Dessau-Roßlau); **Schumacher**, Franz-Josef (Oberhausen); **Schwab**, Robert (Koblenz); **Schwandner**, Thilo (Lich); **Schwarz**, Jochen Günter (Rottenburg); **Schymatzek**, Ulrich (Eitorf); **Spangenberger**, Wolfgang (Bergisch-Gladbach); **Sperling**, Peter (Montabaur); **Staade**, Katja (Düsseldorf); **Staib**, Ludger (Esslingen); **Staikov**, Plamen (Frankfurt am Main); **Stamm**, Ingrid (Heppenheim); **Stark**, Wolfgang (Roth); **Stechemesser,** Bernd (Köln); **Stengl**, Wolfgang (Nürnberg); **Stern**, Oliver (Hamburg); **Stöltzing**, Oliver (Meißen); **Stolte**, Thomas (Mannheim); **Stopinski,** Jürgen (Schwalmstadt); **Stratmann**, Gerald (Goch); **Straßburger**, Harald (Alfeld); **Stubbe**, Hendrik (Güstrow/); **Stülzebach**, Carsten (Friedrichroda); **Tepel,** Jürgen (Osnabrück); **Terzić**, Alexander (Wildeshausen); **Teske,** Ulrich (Essen); **Thasler**, Wolfgang (München); **Tichomirow,** Alexej (Brühl); **Tillenburg**, Wolfgang (Marktheidenfeld); **Timmermann,** Wolfgang (Hagen); **Tomov**, Tsvetomir (Koblenz; **Train**, Stefan H. (Gronau); **Trauzettel**, Uwe (Plettenberg); **Triechelt**, Uwe (Langenhagen); **Ulbricht**, Wolfgang (Breitenbrunn); **Ulcar**, Heimo (Schwarzach im Pongau); **Ungeheuer**, Andreas (München); **Unger**, Solveig (Chemnitz); **Utech**, Markus (Gelsenkirchen); **Verweel**, Rainer (Hürth); **Vogel**, Ulrike (Berlin); **Voigt**, Rigo (Altenburg); **Voit**, Gerhard (Fürth); **Volkers**, Hans-Uwe (Norden); **Volmer**, Ulla (Berlin); **Vossough**, Alexander (Neuss); **Wallasch**, Andreas (Menden); **Wallner**, Axel (Lüdinghausen); **Warscher,** Manfred (Lienz); **Warwas**, Markus (Bonn); **Weber**, Jörg (Köln); **Weber**, Uwe (Eggenfelden); **Weihrauch**, Thomas (Ilmenau); **Weiß**, Heiko (Aue); **Weiß**, Johannes (Schwetzingen); **Weißenbach**, Peter (Neunkirchen); **Werner**, Uwe (Lübbecke-Rahden); **Wessel,** Ina (Duisburg); **Weyhe**, Dirk (Oldenburg); **Wicht**, Sebastian (Bützow); **Wieber**, Isabell (Köln); **Wiens**, Matthias (Affoltern); **Wiesmann**, Aloys (Rheine); **Wiesner**, Ingo (Halle); **Withöft**, Detlef (Neutraubling); **Woehe**, Fritz (Sanderhausen); **Wolf**, Claudio (Neuwied); **Wolkersdörfer**, Toralf (Pößneck); **Yaksan**, Arif (Wermeskirchen); **Yildirim**, Can (Lilienthal); **Yildirim**, Selcuk (Berlin); **Zarras**, Konstantinos (Düsseldorf); **Zeller**, Johannes (Waldshut-Tiengen); **Zhorzel**, Sven (Agatharied); **Zuz**, Gerhard (Leipzig);
